# The serious full-length forearm injury - a case report and literature review

**DOI:** 10.1186/s12891-020-03394-z

**Published:** 2020-06-15

**Authors:** Jun Wang

**Affiliations:** 1 Department of orthopedic, Xiaoshan 1st People’s Hospital, Hangzhou, 311200 China; 2 Department of orthopedic, Xiaoshan 1st People’s Hospital, No. 199 Shixin Road, Hangzhou, 311201 Zhejiang Province China

**Keywords:** Dislocations, Elbow joint, Wrist injuries

## Abstract

**Background:**

Among upper limb injuries, carpal bone fractures and dislocation, Essex-Lopresti injury, and the terrible triad injury of the elbow are serious and relatively rare injuries. These injuries require surgical intervention. The surgical method is difficult, and the treatment effect is poor. These injuries have not been described in the same limb in the literature.

**Case presentation:**

A 21-year-old male patient fell from a height in our institution and sustained multiple injuries, including carpal bone fracture-dislocation, Essex-Lopresti injury, and the terrible triad injury of the elbow of his right upper limb. After 2 surgeries and rehabilitation, he returned to work. We reviewed available reviews and related literature on serious upper-limb damage.

**Conclusions:**

Full-length forearm injury is very rarely encountered, and the management of such fractures is difficult. Radial head replacement with a metal prosthesis, reconstructed the IOM with Tightrope, and fixed the DRUJ with a K-wire pin is appropriate treatment.

## Background

Among upper limb injuries, carpal bone fracture-dislocation is relatively rare, and perilunate dislocations and fracture-dislocations account for approximately 10% of wrist injuries [1].Lunate subluxation, magnum bone, and scaphoid bone fracture are difficult to diagnose. A missed diagnosis can lead to wrist pain, and most of these injuries require open reduction and internal fixation [2]. The Essex-Lopresti injury is rare and caused by a high energy load, and the pattern of the injury consists of a fracture of the radial head (RH), account for 1% radial head fracture. Disruption of distal radioulnar joint (DRUJ) and rupture of the interosseous membrane. Repair of these injuries is technically demanding for surgeons [3]. The terrible triad of the elbow is one of the most challenging injuries due to its complex injury pattern and compromised clinical results. This high-energy injury of the elbow joint consists of a characteristic triad, namely, posterior dislocation of the elbow associated with a radial head fracture and a coronoid fracture [4]. These injuries share the following characteristics: 1. high-energy injury; 2. low incidence; and 3. extreme difficulty to treat. These damages occurred in the same limb, and such a case has not been described in the literature.

## Case presentation

**Fig. 1:**
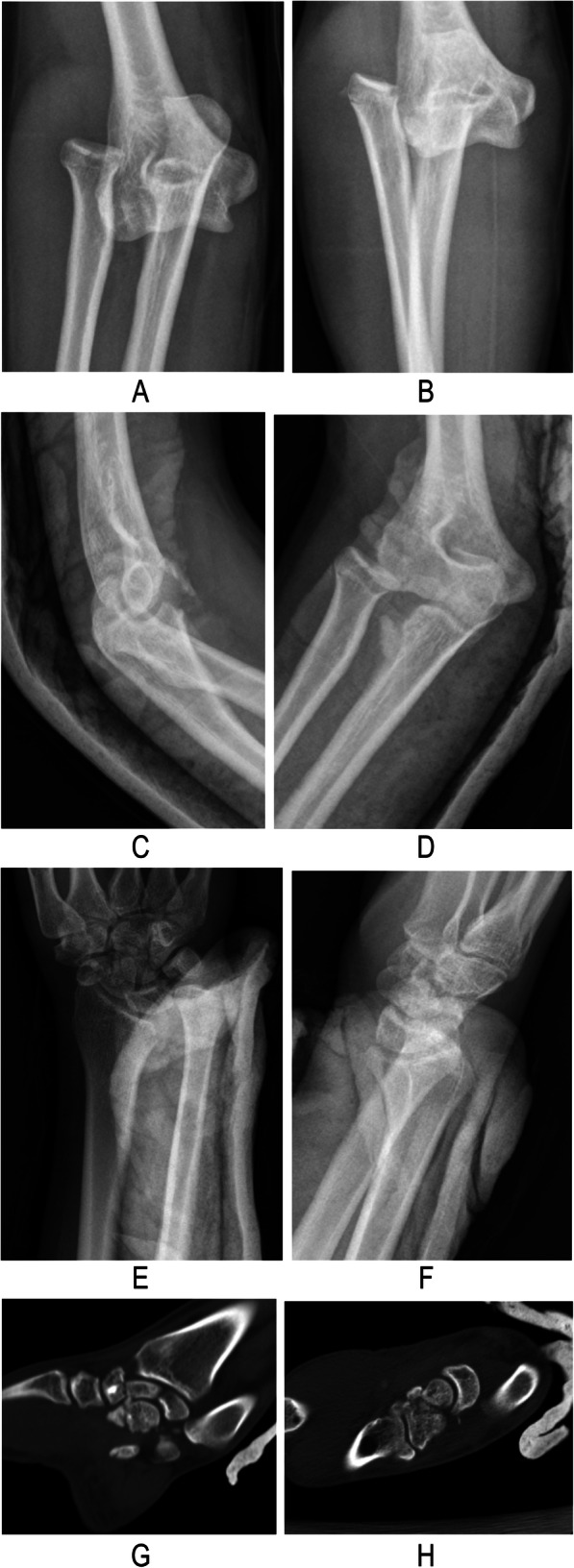
First radiographs, **a** anteroposterior view, **b** oblique view; **c**-**d** elbow joint after reduction; **e**-**f** wrist joint; **g**-**h** wrist joint CT

**Fig. 2:**
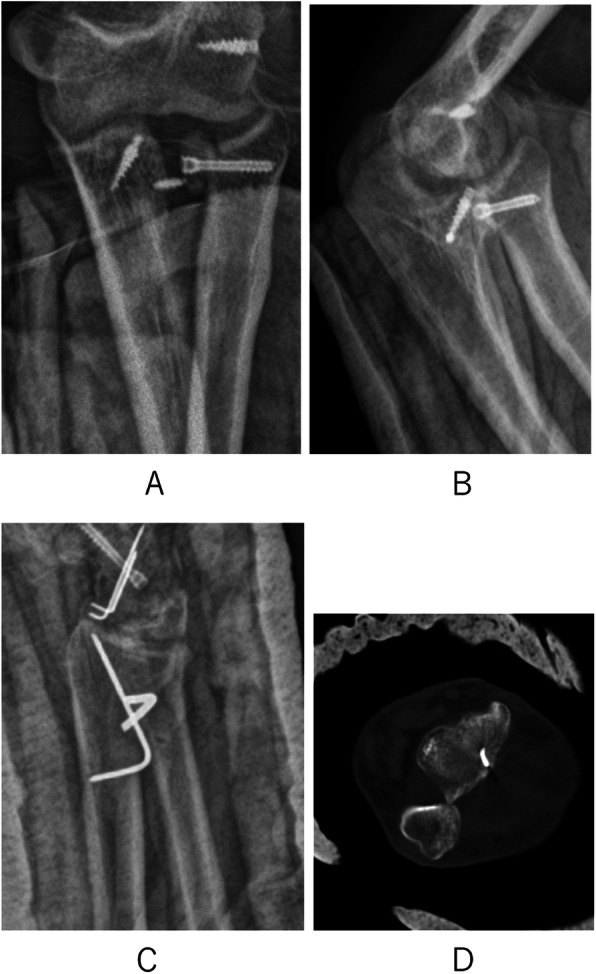
After first surgery radiographs. **a-b**, elbow joint; **c**, wrist joint; **d**, CT: wrist joint

**Fig. 3:**
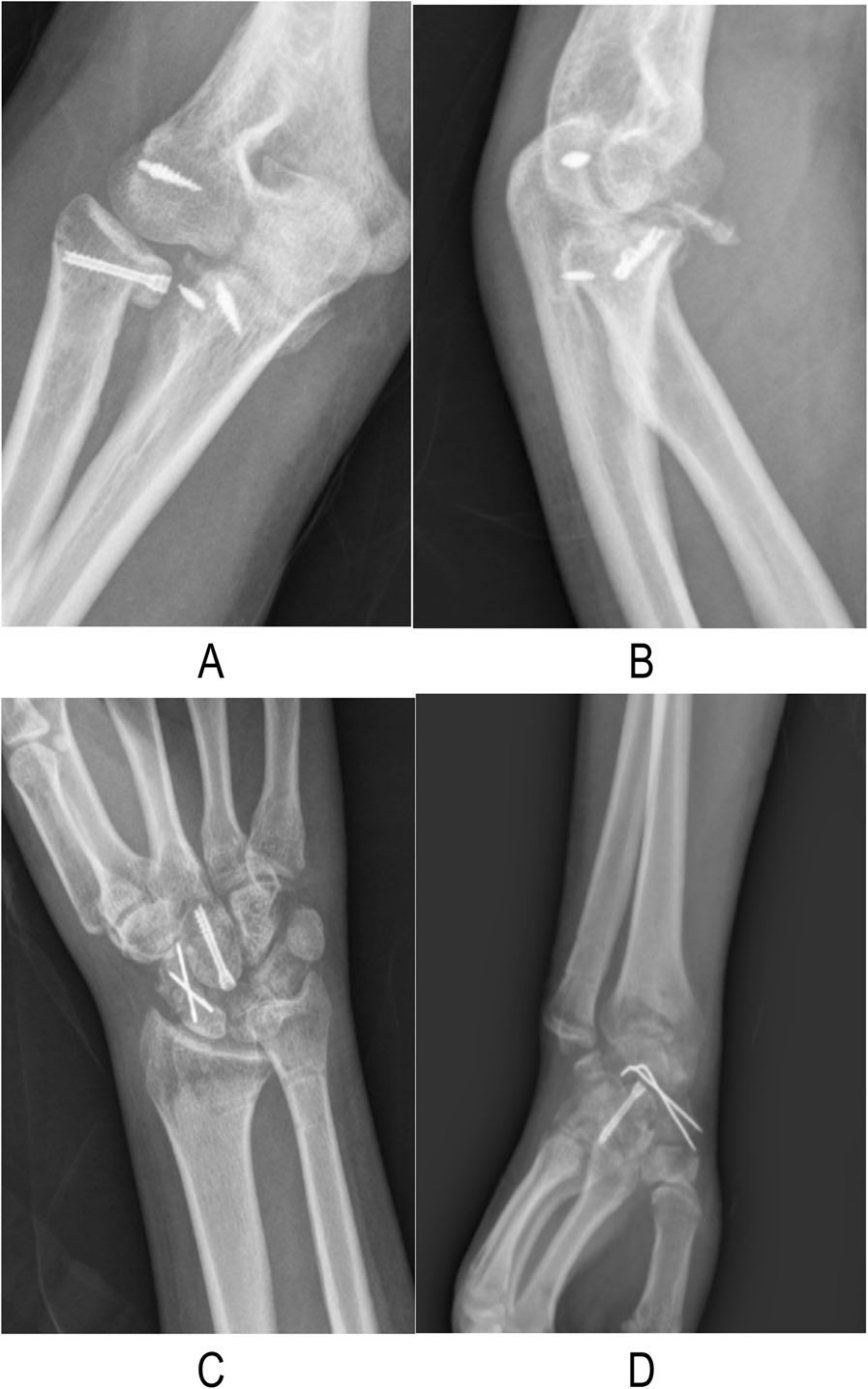
Two months after first surgery radiographs. **a-b**, elbow joint; **c-d**,wrist joint

**Fig. 4:**
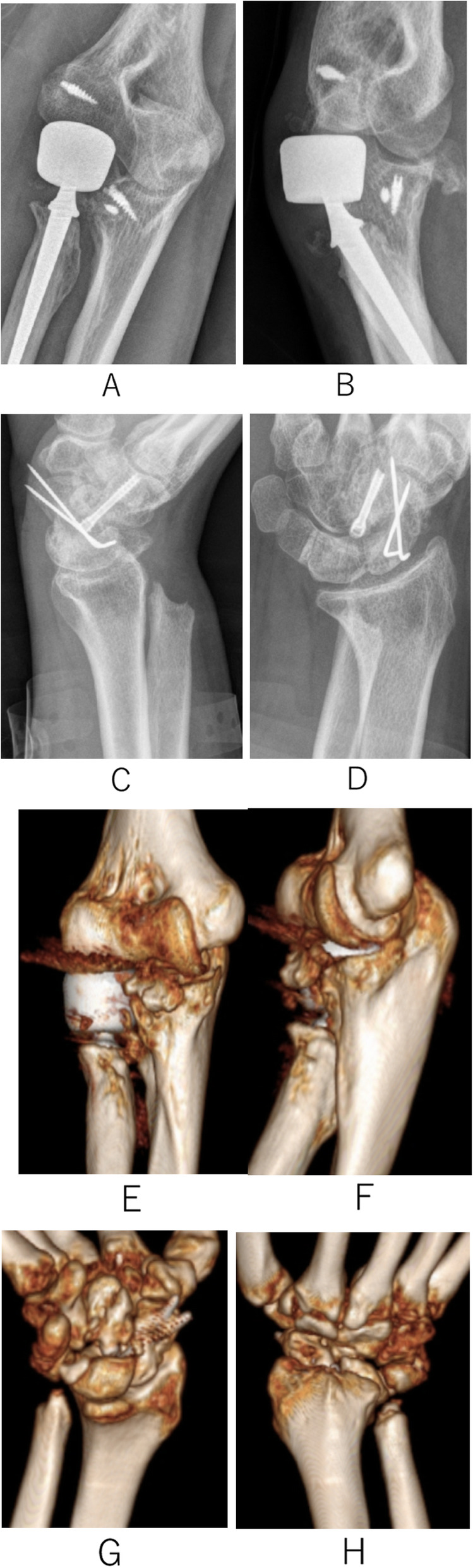
One year after second surgery radiographs. **a-b**, elbow joint; **c-d**, Wrist joint; **e-f**, CT: elbow joint; **g-h**, CT: wrist joint

In November 2017, a 21-year-old man fell 7 m from an operation frame and injured his right upper extremity, lumbar spine, and pelvis. He presented to our institution 2 h after the injury. Physical examination revealed high-grade swelling, pain, deformities and limitation of function of the wrist and elbow as well as pain and limitation of function of the lumbar spine and pelvis. Radiographs of the wrist, elbow, lumbar spine, and pelvis revealed fractures of the magnum bone, scaphoid bone, the radial head, the coronoid, the first lumbar vertebrae, and pelvis. Moreover, dislocation of the distal radioulnar joint, elbow, and lunate subluxation were noted. The diagnosis included carpal bone fracture-dislocation, Essex-Lopresti injury, terrible triad of the elbow (Fig. 1), lumber fracture, and pelvic fracture. He received analgesia for reduction of the elbow dislocation in the emergency room. Seven days later, surgical treatment was performed. Under general anesthesia, we performed the following techniques: open reduction and internal fixation with a k-wire and Hebert screw via a dorsal incision to treat the carpal bone fracture-dislocation; open reduction and internal fixation with a Herbert screw to fix the radial head; suture anchor to fix the ulna coracoid process and radial collateral ligaments of elbow joint to treat the Essex-Lopresti injury and the terrible triad of elbow through an anterolateral approach of the elbow; and K-wire fixation to fix the distal radioulnar joint via a dorsal incision of the wrist (Fig. 2). X-rays show the malposition of the distal radioulnar joint 1 week after the surgery. We removed the K-wire. Two months later, the patient felt pain in his wrist. We found that the malposition of the distal radioulnar joint was unchanged (Fig. 3). After a discussion with his parents, revision surgery was performed. The radial head was replaced with a metal prosthesis, and the distal ulna was removed. (Fig. 4).

**Fig. 5:**
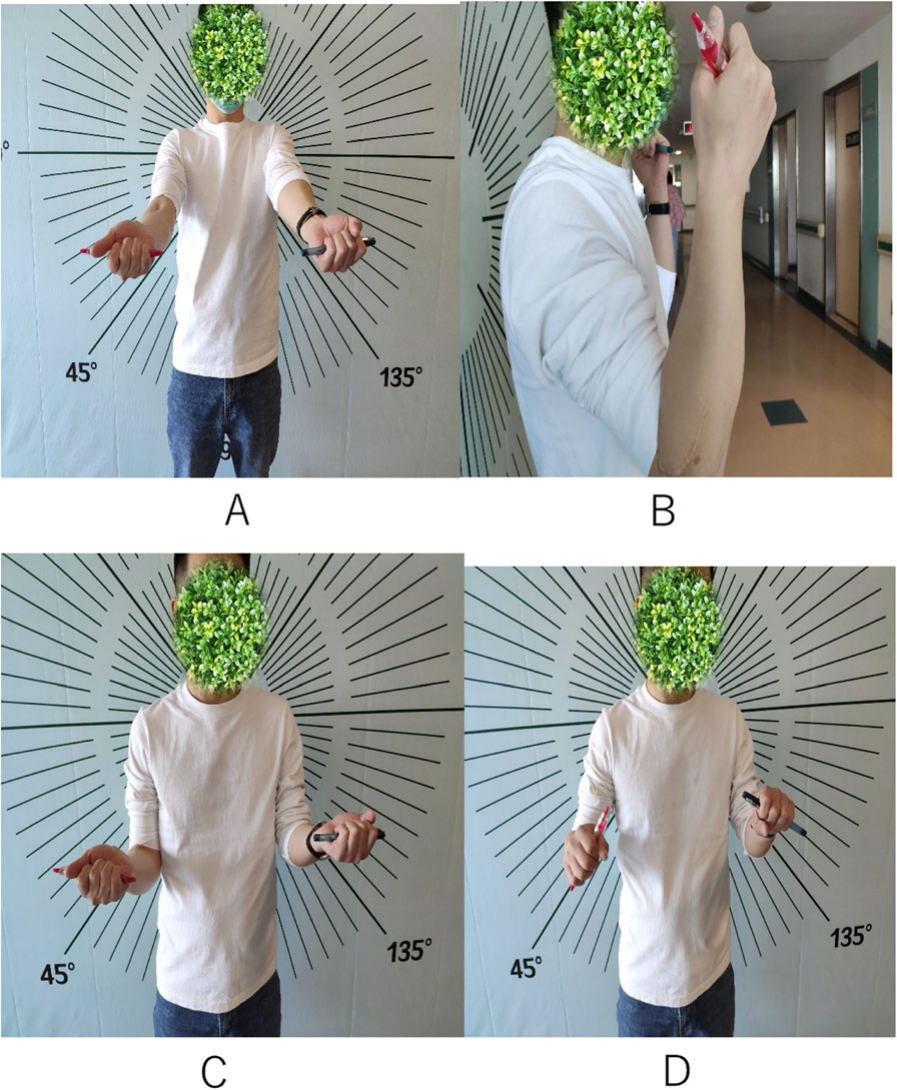
Three years after second surgery photographs. **a-d**, the elbow joint and forearm function

After 12 months, the patient regained elbow flexion and extension strength (range of motion, 10°–130°). His wrist was stable with subluxation of the distal radioulnar joint. The following forearm motion range was observed: supination, 0°–70°; and pronation, 0°–60°. The patient had returned to work. Three years later, we had checked the elbow joint and forearm function.(Fig. 5).

## Discussion and conclusions

The terrible triad of the elbow and the Essex-Lopresti injury are severe and extremely rare upper-limb injuries [5, 6]. We believe it is necessary to analyze the biomechanical relationship between the terrible triad of the elbow and the Essex-Lopresti injury. The injury mechanism for these injuries involves a violent axial injury from the hand to the elbow with pronation or supination of the forearm. The terrible triad injury typically occurs when an axial load is applied to the arm. In addition, the elbow sustains a valgus load, and the forearm sustains supination. Eventual posterior elbow dislocation is associated an impaction fracture of the radial head and shear fracture of the coronoid process [7, 8]. The Essex-Lopresti injury typically results when an axial load is applied to the arm. The radiocapitellar joint has the most contact when the forearm in pronation. The entire load is transmitted through the radius to the radial head, causing migration of the radius and tearing of the interosseous membrane (IOM) [9].

In addition, the position of the forearm in space determines where the majority of the load is located. In pronation, the strain is mainly found in the proximal region, whereas the strain is increased distally in supination. The strain in the central band is significantly increased after excision as the forearm proceeds from a position of supination to pronation [9].

It is important to recognize that the position of the stretched arm at the time of injury will determine the injury pattern. This information is not commonly described in the literature because the forearm cannot undergo pronation and supination simultaneously. If the force is great enough, the forearm can proceed from a position of supination to pronation [10].

Terrible triad injuries are treated via a standardized surgical procedure that involves fixation or replacement of the radial head, coronoid fracture fixation, repair of the lateral collateral ligament, and if necessary repair of the medial collateral ligament. We used this procedure and obtained a satisfactory surgical outcome [11–13].

The treatment of the Essex-Lopresti injury remains controversial. The treatment has three areas of focus: 1. Treatment of the radial head: In the acute phase, the native radial head should be preserved. The most favorable option involves anatomical reconstruction of the radial head with a Herbert screw or plate. If anatomical reconstruction is not possible, replacement should be performed [14]. In the chronic phase, the patient experiences pain and loss of strength at the elbow and/or wrist joint. The radial head of the elbow must be reestablished with a prosthesis [15, 16]. Allograft, metal and silicone implants are available, and the desired clinical outcome is to recover the length of the limbs [17]. Mayhall et al. [18] reported that silicone implants are not suitable in this procedure. Radial head replacement with metallic implants appears to exhibit better outcomes [17].

2. Treatment of the interosseous membrane (IOM): The IOM plays an important role in forearm stability. However, IOM reconstruction in the acute phase is controversial [19]. Only a limited number of clinical studies on IOM reconstruction in patients have been reported. Brin et al. [20] described a case report of acute repair of the IOM using the TightRope device. IOM reconstruction techniques used during the chronic phase have been developed in recent decades. The ideal technique should involve anatomical reconstruction, and many reconstructive options have been reported in the literature [10, 21]. Currently, two surgical methods are available: autogenous or allograft tendon (i.e., pronator teres tendon [22], bone-patellar tendon [23], and flexor carpi radialis autograft [24]) and various synthetic devices (suture-button [25] and Tightrope [26]). Complications include donor site pain and adverse impacts on forearm rotation [27]. Regardless of the method used to reconstruct the interosseous membrane, the original forearm stability cannot be obtained. Most of the above studies were performed on cadaver specimens, so clinical studies should be performed in the future.

3. Treatment of the distal radioulnar joint (DRUJ): In the acute phase, if good stability is observed, the forearm should be fixed for 6 weeks [28]. If the stability is poor, Kirschner wire or screws can be used to fix the ulnar radioulnar joint. TFCC can be simultaneously repaired to increase the stability of the lower radioulnar joint. In the chronic phase, many authors described treatment via ulnar shortening osteotomy [29]. The treatments for ulnar shortening osteotomy include distal ulna resection and shorten osteotomy of ulnar shaft with Locking Compression Plate (LCP) fixation [30].

In summary, full-length forearm injuries (carpal bone fracture-dislocation, Essex-Lopresti injury, and the terrible triad injury of the elbow) are very rarely encountered, and the management of such injuries can be technically demanding. We should be aware of the principles of the components of the severe injury. In the first surgery for this case, we should have performed radial head replacement with a metal prosthesis, reconstructed the IOM with Tightrope, and fixed the DRUJ with a K-wire pin given the significant force of these injuries. However, we lacked therapeutic experience when the case was first presented. In the second surgery for this case, we adhered to the treatment principles of a chronic Essex-Lopresti injury.

## Data Availability

This is a case report of a single patient, to protect privacy and respect confidentiality; none of the raw data has been made available in any public repository. The original reports, imaging studies and outpatient clinic records are retained as per normal procedure within the medical records of our institution.

## Abbreviations

IOM: Interosseous membrane
DRUJ: Distal radioulnar joint
RH: Radial head
